# Use of Coiled-Coil Affinity Peptides to Manufacture Antibody Conjugates

**DOI:** 10.1021/acs.bioconjchem.5c00178

**Published:** 2025-07-29

**Authors:** Seyed Farzad Baniahmad, Alina Burlacu, Laurence Delafosse, Mauro Acchione, Miriam Simmons, Binbing Ling, Umar Iqbal, Maria J Moreno, Gregory De Crescenzo, Yves Durocher

**Affiliations:** a Department of Biochemistry and Molecular Medicine, Faculty of Medicine, Université de Montréal, Montréal, H3C 3J7 Québec, Canada; b Human Health Therapeutics Research Centre, Building Montreal-Royalmount, 6356 National Research Council Canada, Montréal, H4P 2R2 Québec, Canada; c Human Health Therapeutics Research Center, 6356 National Research Council Canada, Ottawa, K1A 0R6 Ontario, Canada; d Department of Chemical Engineering, 5596 Polytechnique Montréal, Montréal, H3C 3A7 Québec, Canada; e PROTEO: The Quebec Network for Research on Protein Function, Structure, and Engineering, Université du Québec a Montréal, Montréal, H3C 3P8 Québec, Canada

## Abstract

Antibody-drug conjugates are revolutionizing cancer treatment. However, their manufacturing still requires improvements in conjugation technology, especially for the control of the drug-to-antibody ratio (DAR). Here, we investigate the use of the de novo designed coiled-coil heterodimer, composed of the Ecoil and Kcoil peptides, as a new strategy for generating antibody conjugates with high homogeneity and a controllable DAR. More precisely, we investigated the assembly, stability, and tumor targeting of two conjugated antibodies made of (1) trastuzumab with C-terminal Ecoils (TZM-Ecoil) noncovalently paired with Kcoil peptides fused to the monomeric red fluorescent protein (Kcoil-mRFP), yielding TZM-E/K-mRFP or (2) TZM-Ecoil noncovalently paired to Kcoil peptide covalently linked to the fluorescent dye CF750 (Kcoil-CF750), yielding TZM-E/K-CF750. Results from the *in vitro* stability assessment of these complexes in blood serum revealed that their integrity was maintained. Furthermore, *in vivo* biodistribution and tumor localization data using a HER2-expressing SKOV3 xenograft mouse model indicated efficient tumor targeting and retention for up to 10 days postinjection of the TZM-E/K-CF750 conjugate.

## Graphical abstract

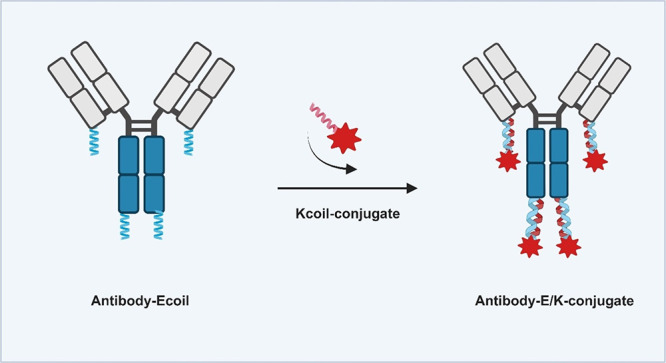

## Highlights

- E/K coiled-coil effectively mediate noncovalent assembly of antibody conjugates with high homogeneity.
- The addition of the Ecoil peptide sequences to the antibody heavy and light chain C-termini allow the generation of antibody conjugates with “Drug-Antibody Ratio” (DAR) of 4.
- Coiled-coil trastuzumab complexed with Kcoil conjugated to the fluorescent dye CF750 effectively reaches the tumor site in a HER2-expressing SKOV3 xenograft mouse model and remains at the tumor site for up to 10 days.

## Introduction

Antibody-drug conjugates (ADC) have emerged as a promising class of targeted cancer therapies. They represent the therapeutic legacy of the ″magic bullet″ concept.^1^ By a combination of the specific targeting properties of monoclonal antibodies with a conjugated cytotoxic payload, ADCs enable the selective delivery of potent chemotherapeutic agents to cancer cells. This targeted approach significantly reduces off-target toxicities compared to traditional chemotherapy, and is expected to result in a wider therapeutic window of anticancer drugs with a lower minimum effective dose and an increased maximum tolerated dose.^2,3^ Although the ADC concept dates back many decades, the successful design and development of these therapeutics remain challenging today. Quality attributes of ADCs such as aggregation, *in vivo* stability, purity, potency, immunogenicity, and batch-to-batch consistency are often difficult to control, due to the biophysical characteristics of the antibody, cytotoxic drug, selected linker, conjugation chemistry, and the manufacturing process.^4–7^

First-generation ADCs primarily relied on chemical conjugation to attach an activated functional group from the linker or linker-payload complex to the antibody’s solvent-accessible side chains of lysine^8^ or cysteine^9^ residues. The majority of FDA-approved ADCs are produced using this approach.^10,11^ However, poor therapeutic index of these early designs due to many factors such as high Drug-to-Antibody Ratio (DAR), product heterogeneity, negative impact of conjugation on antibody functionalities, and reversibility of conjugation reactions, prompted the development of next-generation ADCs, incorporating more precise and controllable conjugation strategies to improve manufacturing consistency, therapeutic efficacy, safety and overall clinical outcomes.^12–14^

To that end, site-specific conjugations targeting engineered amino acids^15–18^ or specific protein sequences^19,20^ on the antibody backbone have allowed for a more precise conjugation at predetermined sites, leading to better control of the DAR and reduced heterogeneity of the end-product. One of these approaches relies on the use of the LCxPxR short peptide sequence to introduce a specific conjugation site on the antibody, followed by treatment with the formylglycine-generating enzyme (FGE).^21,22^ This allows for site-specific biorthogonal conjugation, which is achieved by reacting the resulting aldehyde moiety with the payload of interest, which is equipped with a hydrazine or alkyl amine nucleophilic group to yield a stabilized Schiff base. Drake et al. showed that incorporation of these engineered aldehyde tags at the C-termini of trastuzumab’s (TZM) heavy chains allowed for the conjugation of the cytotoxic maytansine payload (DM1) and resulted in an ADC with improved *in vivo* potency and reduced toxicity compared to the conventional lysine-conjugated TZM-DM1.^23^ Even though the incorporation of tags to antibodies generally improves ADC manufacturability, various challenges remain, such as reduced antibody titers and the potential for immunogenic responses, all of which need to be addressed in order to design more robust and safer ADC platforms.^24,25^ Despite these improvements, over 30 site-specific ADC clinical trials have been discontinued, and no site-specific conjugated ADC has yet received FDA approval.^26^

In this article, we explored an alternative approach for site-specific conjugation that relies on the use of *de novo* designed coiled-coil affinity peptides to tether a payload to the antibody backbone. Among coiled-coil platforms, the five-heptad-long Ecoil ((EVSALEK)_5_ sequence, or E5) and Kcoil ((KVSALKE)_5_ sequence, or K5) peptides have been extensively studied for dimerization and tethering purposes. These two distinct peptides form a parallel heterodimer with high specificity, affinity, and stability under physiological conditions.^27–30^ We previously examined the manufacturability and characteristics of Ecoil-tagged TZM (TZM-Ecoil) for controlled release from hydrogels containing covalently linked Kcoil peptides and found that addition of Ecoil sequence, regardless of their length (i.e., number of heptad repeats) and position at the C-terminus of either the antibody heavy or light chain, did not alter antibody binding affinity and functionality when tested on HER2-expressing cells. In the present study, we evaluated the use of the E/K peptide interactions to develop an antibody conjugate platform in which a Kcoil peptide linked to a surrogate payload molecule (as a fusion to the N-terminus of the monomeric red fluorescent protein; mRFP) was tethered to TZM-Ecoil. We evaluated TZM-Ecoils and Kcoil-mRFPs with 5, 4, or 3 heptad repeats for their ability to form stable coiled-coil complexes, as evaluated by size-exclusion chromatography, while the E/K coiled-coil binding affinities were assessed in a surface plasmon resonance (SPR)-based binding assay. In addition, we evaluated the stability of these surrogate antibody conjugate complexes in blood serum *in vitro* using an enzyme-linked immunosorbent assay (ELISA). We finally monitored tumor accumulation of a TZM-E5/K5-CF750 dye conjugate in a HER2-expressing SKOV3 xenograft mouse model. Despite an observed premature but partial payload release *in vivo*, highlighting the need for further stabilization of the coiled-coil interaction, our results demonstrate the great potential and simplicity of this site-specific antibody conjugation approach, offering a promising platform for future ADC manufacturing.

## Materials and Methods

For clarity, Ex and Kx will refer to the peptides corresponding to the (EVSALEK)_*x*_ and (KVSALKE)_*x*_ sequences, respectively, *x* being equal to 5, 4, or 3.

### 3.1 Expression Plasmids, Production, and Purification of Peptide-Tagged Recombinant Proteins

#### 3.1.1 Trastuzumab Cloning, Purification, and Characterization

Trastuzumab (TZM) heavy chain (HC) and light chain (LC) cDNA with Ecoil sequences (E5, E4, or E3) fused at their C-termini (HC-Ex and LC-Ex) were cloned into the pTT5 expression vector and produced by transient gene expression in CHO cells, then purified, and characterized based on previously described protocols.^31^

#### 3.1.2 Monomeric Red Fluorescent Protein with N-Terminal Kcoil

The cDNA sequence encoding the monomeric red fluorescent protein (mRFP) with the K5 peptide preceded by the IGFBP1 signal peptide (MSEVPVARVWLVLLLLTVQVGVTA) at its N-terminus was synthesized by Genscript and cloned into the pTT5 plasmid. The resulting plasmid is referred to as pTT5-SPK5mRFP. This plasmid was double-digested with *Eco*RI and *NheI* restriction enzymes to replace the SPK5 fragments with DNA sequences encoding SPK4 or SPK3 peptides, resulting in SPK4mRFP and SPK3mRFP constructs, respectively. All mRFP constructs were designed to contain a C-terminal 8xHis tag for purification purposes.

All secreted Kcoil-tagged mRFPs were produced by transient gene expression (TGE) in CHO-3E7 cells based on published methods.^32,33^ The cultures were harvested at 6 days post-transfection (viability > 70%), and supernatants were clarified by centrifugation (40 min, 4000*g*) and sterile-filtered using a 0.22 μm membrane filter (Express PLUS, Millipore). Subsequently, immobilized metal affinity chromatography (IMAC) was performed using nickel sepharose excel resin (GE Healthcare) to purify the mRFP constructs. Following column equilibration with 50 mM NaH_2_PO_4_ pH 7.0, 300 mM NaCl, supernatants were loaded at 1 mL/min, and columns were then washed once with five column volumes of 50 mM NaH_2_PO_4_ pH 7.0 containing 300 mM NaCl and 10 mM imidazole. Bound proteins were eluted with elution buffer (50 mM NaH_2_PO_4_, pH 7.0, 300 mM NaCl, and 300 mM imidazole). The fractions containing eluted Kcoil-tagged mRFPs were pooled, and the elution buffer was exchanged for phosphate-buffered saline (PBS) using CentriPure P-25 desalting columns (emp Biotech GmbH, Germany).

#### 3.1.3 Trastuzumab-Monomeric Red Fluorescent Protein (TZM-mRFP) Fusion

The mRFP cDNA was fused in-frame to the C-termini of TZM HC and TZM LC sequences using a 28 amino acid-long linker. The resulting plasmids, pTT5-TZMHC-mRFP and pTT5-TZMLC-mRFP, were used for the production of the TZM-mRFP fusion antibody as described in Section 3.1 with minor modifications. Briefly, 24 h before transfection, CHO-3E7 cells were seeded in FreeStyle F17 medium (Invitrogen) supplemented with 4 mM glutamine (Sigma-Aldrich) and 0.1% Kolliphor P188 (Sigma-Aldrich). The following day, cells (at a density of 2.0–2.2 × 10^6^ cells/mL) were transfected with a DNA mixture containing 40% (w/w) pTT5-TZMHC-mRFP, 40% pTT5-TZMLC-mRFP, 5% pTTo-GFP, and 15% pTT22-hAktDD. Following the incubation of the DNA mixture and polyethylenimine (PEImax, PolySciences) at a 1:4 ratio, the mixture was added to cells, and flasks were incubated under constant agitation (120 rpm) at 37 °C under a humidified atmosphere containing 5% CO_2_. Protein-A purification of the antibody constructs from the harvested clarified supernatants (typically at day 6 to 8 post-transfection) was performed as previously described.^34,35^

#### 3.1.4 Protein Characterization and Quantification

As described previously,^31^ we characterized all proteins using sodium dodecyl sulfate–polyacrylamide gel electrophoresis (SDS-PAGE) and ultraperformance liquid chromatography (UPLC) size-exclusion chromatography–multiangle light scattering (SEC-MALS). TZM-Ecoil and TZM-mRFP fusions were quantified by protein-A HPLC using an 800 μL POROS 20 μm protein-A ID Cartridge (Applied Biosystems) according to the manufacturer’s recommendations. Kx-mRFP products were quantified by absorbance at 280 nm using a Nanodrop spectrophotometer (ThermoFisher Scientific) and the calculated extinction coefficient for each protein (ProtParam tool – Expasy).

### 3.2 Chemical Conjugation

The K5 peptide, exhibiting a C-terminal cysteine, i.e., (KVSALKE)_5_-GGC, was synthesized by solid phase peptide synthesis (SPPS; Sherbrooke University). Aliquots were prepared in Milli-Q water and stored at −80 °C. CF-750 (CF750) maleimide near-infrared dye (96062) and CF750 Dye SE/TFP ester (92142) were purchased from Biotium (Hayward, CA), while 2,4,6-trinitrobenzenesulfonic acid (TNBS), 1% in methanol, was purchased from G-Biosciences (St. Louis, MO) and stored at −20 °C.

DTNB (5,5-dithio-bis-[2-nitrobenzoic acid]; Ellman’s Reagent) and TCEP (Tris­(2-carboxyethyl) phosphine) reducing agent were purchased from ThermoFisher Scientific and stored according to the manufacturer’s recommendations. Endosafe water was purchased from Charles River Laboratories, and an Amicon ultracentrifugal filter (Cut-off 3 kDa MW) was purchased from Millipore, ED. All the other chemical reagents were purchased from Sigma-Aldrich and stored according to the manufacturer’s recommendations.

#### 3.2.1 CF750-Tagged Kcoil Peptide

The (KVSALKE)_5_-GGC peptide was conjugated to CF750 maleimide near-infrared (NIR) dye. Prior to the conjugation reaction, the accurate peptide concentration and level of oxidation, using TNBS and the Ellman assay, were assessed based on previously described protocols.^36–38^ (KVSALKE)_5_-GGC (206 nmol; 800 μL of a 260 μM solution) was incubated with 2 μmol of TCEP (4 μL of a 0.5 M solution) reducing agent for 2 h in the dark. Then the mixture was buffer exchanged in Endosafe water using an Amicon ultra centrifugal filter (Cut-off 3 kDa MW), applying a flushing procedure described by the manufacturer to remove unreacted TCEP. Immediately after, the mixture was combined with 206 nmol CF750 maleimide dye (20.6 μL of a 10 mM solution) in the presence of nitrogen gas and incubated overnight at room temperature. Determination of the degree of labeling was performed by LC-MS intact mass analysis on a LTQ-Orbitrap XL using the myoglobin tune system.

#### 3.2.2 CF750-Tagged TZM

TZM in phosphate buffer saline (PBS, pH 7.4) was supplemented with sodium bicarbonate buffer, pH 9.3 (10% v/v), to achieve a solution pH of 8.0. To this mixture, a 6-fold molar excess of near-infrared CF750 monoreactive NHS-ester in dimethyl sulfoxide (DMSO) was added and allowed to react at room temperature for one h with slow mixing, followed by an overnight incubation at 4 °C. Labeling was optimized to obtain a dye/antibody ratio of ∼3.5 to 4. After the incubation period, the TZM-CF750 conjugate was freed from unreacted material and buffer-exchanged into PBS, pH 7.4, using an Amicon ultra centrifugal filter (cut-off 3 kDa MW), and the dye-to-protein ratio was calculated by measuring the absorbance at 280 nm (protein) and 750 nm (dye) using a Nanodrop spectrophotometer (Thermo Fisher Scientific).

### 3.3 *In Vitro* Assays

#### 3.3.1 Ultraperformance Liquid Chromatography Size-Exclusion Chromatography-Multiangle Light Scattering (UPLC-SEC-MALS) Analysis

TZM-Ecoil molecules, each with the same number of heptad repeats (thereafter denoted x) in the Ecoil moieties of their heavy (H) and light (L) chains (HEx and LEx, respectively, leading to TZM-Ex constructs), were mixed with Kcoil-mRFP (also harboring x heptad repeats, and thereafter denoted Kx-mRFP) at a 1:4 molar ratio (i.e., 2 nmol of TZM-Ex mixed with 8 nmol of Kx-mRFP; *x* = 3, 4, or 5). Samples were incubated for 45 min at room temperature and subjected to UPLC-SEC-MALS analysis using a 4.6 × 150 mm BEH200 SEC column with 1.7 μm particle size (Waters, Milford, MA) connected to an Acquity H-Class Bio UPLC system (Waters) with a photodiode array (PDA) detector. Chromatography was performed in a mobile phase (0.2 M potassium phosphate, 0.2 M potassium chloride, 0.02% Tween-20, pH 7.0) at 30 °C at a flow rate of 0.4 mL/min. Measurement of the integrated areas and determination of the retention time (RT) for peaks at 280 nm were performed by using Empower 3 software (Waters, Milford, MA). Multiangle light scattering (MALS) data and refractive index (RI) data were collected on Wyatt microDAWN and Wyatt Optilab UT-rEX detectors, respectively. *M*_MALS_ (weighted average molecular mass) was calculated in ASTRA 8 software using the protein concentration determined from the RI signal using a d*n*/d*c* value of 0.185.

#### 3.3.2 Surface Plasmon Resonance-Based Binding Assay

Surface plasmon resonance (SPR) assays were performed on a Biacore T100 biosensor using CM5 sensor chips (Cytiva). Buffers, sensor chip surface functionalization, sensograms acquisition, and data analysis were carried out as previously published.^31^ To study the interaction of TZM-Ecoil with their Kcoil counterpart, 15–40 resonance units (RU) of each cysteine-tagged Kcoil peptide (i.e., (KVSALKE)_*x*_-GGC where *x* = 3, 4, or 5) were immobilized on the sensor surfaces. The remaining reactive groups were then blocked using l-cysteine, on both the test surface and the reference/control surface. Each TZM-Ecoil in HBS-EP (0.01 M HEPES pH 7.4, 0.15 M NaCl, 3 mM ethylenediamine tetraacetic acid, and 0.005% v/v Tween-20 surfactant) was injected at various concentrations on both surfaces at 50 μL/min for 250s, followed by 3600s of HBS-EP only. Surfaces were regenerated by injecting three 20 s pulses of 6 M guanidine hydrochloride. All injections were performed in duplicates at 37 °C.

#### 3.3.3 Serum Stability Analysis

Black 384-well plates with an optically clear flat bottom (ThermoFisher Scientific) were used in this assay. HER2 antigens were produced by transient gene expression (TGE) in CHO-3E7 cells^32,33^ and purified by IMAC on nickel sepharose excel resin (GE Healthcare), as described in Section 3.2. Bovine serum albumin (Millipore Sigma) 1% (w/v) in Dulbecco’s phosphate-buffered saline (Hyclone) was used as a blocking buffer. Tween-20 (BioRad) 0.05% (v/v) solution in PBS was used as a wash buffer. A 1% BSA/0.1% Tween-20 in PBS solution was used as assay/dilution buffer. Antibiotic–antimycotic reagent was purchased from ThermoFisher Scientific and added to all buffers to avoid bacterial and fungal contamination during the experiment. Fetal bovine serum was purchased from ThermoFisher Scientific. Rabbit anti-RFP antibody was purchased from Rockland Immunochemicals.

Plates were coated with 25 μL per well of HER2 antigen (5 μg/mL) and incubated overnight at 2–8 °C. The day after, plates were washed with 50 μL washing buffer and blocked with 50 μL blocking buffer for 1 h at room temperature. Two nmol of TZM-Ecoil were mixed with 8 nmol of Kcoil-mRFP as described in section 2.3.1, followed by 45 min incubation at room temperature. Serial dilutions (1:2 dilutions giving 8 concentrations from 2.8 to 0.02 ng/μL) of each mix were prepared in the dilution buffer, and 25 μL/well of each dilution was transferred to the plate. For the positive control, the same concentration range (2.8 to 0.02 ng/μL) of TZM-mRFP fusion protein was prepared in the assay buffer, and 25 μL/well of each dilution was added to the plate. Plates were incubated on a shaker (250 rpm) at room temperature for 1 h. After a wash step, 50 μL of FBS or PBS was added to the wells. Plates were incubated for 24, 48, and 72 h at 37 °C. Plates were then washed 4 times with 50 μL of wash buffer, and 20 μL of rabbit anti-RFP antibody (1:1000 v/v dilution) was added to each well and incubated for 1 h at room temperature. Finally, 25 μL each of TMB solution was added to each well, and following a 30 min incubation at room temperature, stop solution (H_2_SO_4_) was added to complete the assay. The plates were read at 450 nm with a reference set to 620 nm with a BioTek Cytation 5 microplate reader. Experiments were carried out in triplicate, and data interpretation and representative EC_50_ values were calculated using GraphPad Prism v8 software with a four-parameter logistic model.

### 3.4 *In Vivo* Studies

#### 3.4.1 Animal Studies

The animal experiments were carried out in strict accordance and compliance with the Canadian Council on Animal Care, under protocols that were approved by the Animal Care Committees of the NRC. Female SKH1 hairless immunocompetent albino (SKH1-Hrhr, Strain code 686) and female athymic nude/nude immunodeficient (NU­(NCr)-Foxn1nu Strain code 490) mice were obtained from Charles River Laboratories, (Hollister, CA, USA) and housed in ventilated cages in a pathogen-free, environment-controlled room at 19–21 °C, with a relative humidity ranging between 40% and 70%, a photoperiod of 12 h light and 12 h darkness, and food and water provided *ad libitum*. To evaluate the biodistribution of TZM-E5/K5-CF750 coiled-coil conjugate versus covalently conjugated TZM-CF750, we designed two studies: a first using a nonxenograft, immunocompetent SKH1 mouse model to study the general biodistribution of the antibody conjugates and a second using a SKOV3 xenograft mouse model to study their distribution and tumor accumulation.

Athymic Nu/Ncr mice (4–6 week-old) were subcutaneously implanted with 5 × 10^6^ SKOV3 ovarian cancer cells in the left flank under anesthesia (4–5% isoflurane). Tumors were allowed to grow until they reached an average volume of ∼300 mm^3^, at which point the antibody conjugate was intravenously (IV) administered, as previously described.^39^

Mice from both strains, SKH1 Elite and nude mouse bearing SKOV3 tumor, were divided into three groups (*n* = 3 mice per group) with an additional naïve animal included for organ collection. Animals were fed an alfalfa-free diet to reduce autofluorescence background during imaging.

Three samples were tested in this experiment: TZM-E5 complexed with K5 chemically conjugated to CF750 dye (group 1: TZM-E5/K5-CF750), TZM chemically conjugated to CF750 (group 2: TZM-CF750), and E5 peptide complexed with K5 chemically conjugated to CF750 dye (group 3: E5/K5-CF750). Equimolar amounts of TZM-E5/K5-CF750 (by mixing TZM-E5 with K5-CF750 in a 1:4 molar ratio), TZM-CF750, and E5/K5-CF750 (by mixing E5 with K5-CF270 in a 1:1 molar ratio) were prepared and thoroughly mixed by pipetting up and down and kept at room temperature for ∼45 min prior to administration.

Animals received a single IV injection via the tail vein. Group 1 (TZM-E5/K5-CF750) and group 2 (TZM-CF750) were administered at a final dose of 10 mg/kg, while group 3 (E5/K5-CF750) was injected at a dose of 2.6 mg/kg to match the molar concentration of the TZM conjugates. Fluorescence imaging (dorsal and ventral) was performed at various time points to determine the *in vivo* biodistribution and tumor accumulation of the CF750 dye complexes. Blood was collected from the submandibular vein in heparinized tubes at the end of the study and stored at 4 °C, pending analysis. At the scheduled terminal end point (336 h postinjection), mice were euthanized by cardiac puncture-induced exsanguination while under anesthesia, followed by heparinized saline perfusion. Organs (brain, heart, lung, liver, kidneys, and spleen) were excised and imaged ex vivo.

#### 3.4.2 Imaging Studies

All fluorescence images were obtained at wavelengths of 740 nm (excitation) and 790 nm (emission) by using an IVIS Lumina III preclinical animal imager (PerkinElmer, Waltham, Massachusetts, USA). Total fluorescence radiance efficiency was determined from the whole body and region of interest (ROI) using the Living Image 4.1 software (PerkinElmer, Waltham, Massachusetts, USA).

## Results

### 4.1 Production, Purification, and Manufacturability of the Ecoil-Fused Antibodies and Kcoil-Fused mRFPs

In this study, we present a novel method for tethering a payload to an antibody moiety without the need for chemical or enzymatic modification by using the E/K coiled-coil dimerization system. Our previous research^31^ demonstrated that the incorporation of Ecoil peptide sequences (EVSALEK)_*x*_, regardless of their length (*x* = 3, 4, or 5) and position (C-termini of light and/or heavy chains), has minimal impact on the expression levels of these antibody fusions in Chinese hamster ovary (CHO) cells compared to the wild-type TZM construct.^31^ We now investigate the application of these Ecoil-fused antibodies to establish an antibody conjugate platform. As a proof-of-concept, we employ fluorescent tags (mRFP or CF750) linked to the complementary Kcoil peptides as the payload molecules.

We first designed three Kcoil peptides (consisting of 3, 4, and 5 heptad repeats) fused to the N-terminus of the monomeric red fluorescent protein (mRFP), which serves as a surrogate payload. These fusion protein constructs were all preceded by a signal peptide to allow secretion in the culture medium and were expressed in CHO cells. The yields and purity obtained from a 250 mL production volume of the different mRFP constructs after IMAC purification are shown in Figure S1. As expected, SDS-PAGE analysis of the final chimeric products indicated a size difference in the chimeric proteins corresponding to the presence and length of the Kcoil tags when compared to the mRFP control, under both reducing and nonreducing conditions (Figure S1, panel B). The migration pattern of K5-mRFP (lanes 2 and 6), K4-mRFP (lanes 3 and 7), and K3-mRFP (lanes 4 and 8) aligned with their respective molecular mass differences due to the length of their coil moiety, indicating that Kcoils were present in all products. In addition to the main bands, three additional minor bands were observed in both nonreduced and reduced mRFP products. These bands are likely artifacts originating from the SDS-PAGE sample preparation process, which was previously described to cause partial site-specific hydrolysis of the mRFP protein.^31,40^ This partial cleavage of mRFP is, however, unlikely to happen under physiological conditions.

### 4.2 Coiled-Coil-Mediated Complex Formation Analysis by UPLC-SEC

The interactions between the TZM-Ecoil and Kcoil-mRFP constructs were investigated by UPLC-SEC analysis. Here, the use of Kcoil-tagged mRFP constructs allowed for a robust assessment of their complexation with Ecoil-tagged TZM by monitoring changes in their elution time from the SEC column.

The TZM-Ex constructs (harboring a total of four Ecoil peptides) were mixed with their Kx-mRFP counterparts (with *x* equal to 3, 4, or 5) in a 1:4 molar ratio. The mixes were incubated at room temperature for 45 min before injection on the UPLC-SEC column, and TZM-Ecoil and Kcoil-mRFP were injected individually, as controls. In addition, wild-type TZM (Figure 1A: blue line; elution at 3.06 min) and the TZM-mRFP fusion (Figure 1A: black line; elution at 2.54 min) constructs were also included as controls. Figure 1B shows overlaid chromatograms of TZM-E5 (blue line), K5-mRFP (red line), and their mixture (black line). The mixture of both proteins (black line) resulted in the appearance of a distinct, faster eluting peak (i.e., of higher relative molecular mass, Mr) at 2.41 min, while the peaks corresponding to TZM-E5 (2.66 min) and K5-mRFP (3.37 min) disappeared, indicating complex formation through coiled-coil interactions. Additionally, multiangle light scattering (MALS) analysis of the peak at 2.41 min revealed a molecular weight (338 kDa; Table S1) consistent with the presence of one TZM-E5 and four K5-mRFP molecules, further supporting a 1:4 stoichiometry for the coiled-coil assembled complex.

**Figure 1:**
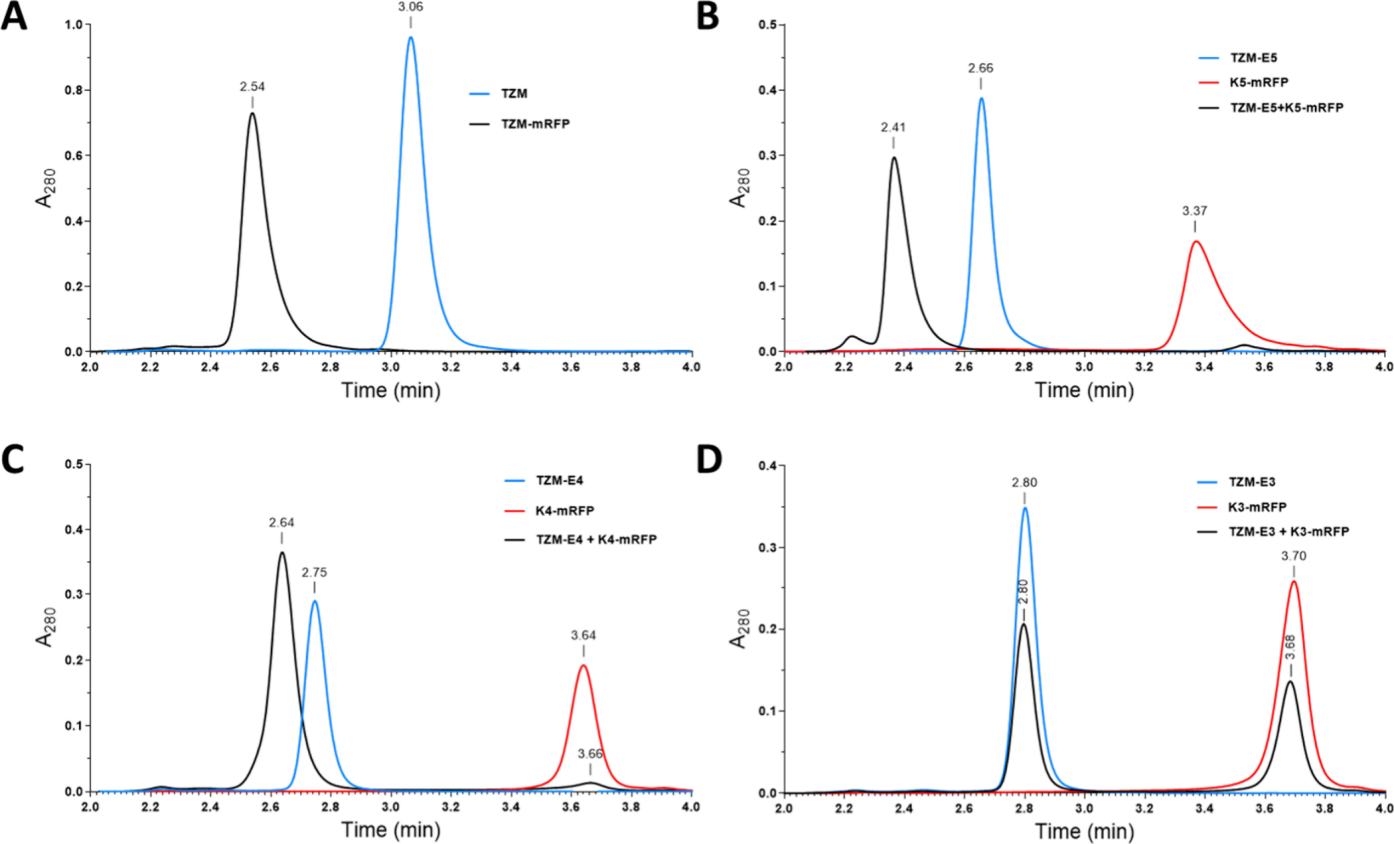
UPLC-SEC chromatograms for TZM, TZM-mRFP, TZM-Ex, Kx-mRFP, and their mixes: (A) TZM (blue line) and TZM-mRFP (black line); (B) TZM-E5 (blue line), K5-mRFP (red line), and their mixture in 1:4 ratio (black line); (C) TZM-E4 (blue line), K4-mRFP (red line), and their mixture in 1:4 ratio (black line); and (D) TZM-E3 (blue line), K3-mRFP (red line), and their mixture in 1:4 ratio (black line). Individual proteins or their mixtures were incubated for 45 min at room temperature before being injected on the UPLC-SEC column.

TZM-E4 mixed with K4-mRFP gave similar results (Figure 1C). In stark contrast, no coiled-coil mediated complex formation was observed when TZM-E3 was mixed with K3-mRFP (Figure 1D). This finding is consistent with our previously published data indicating that E3/K3 peptide dimers are of low affinity and lack stability.^28,31,41^

Additional experiments aimed at investigating complex stability upon dilution and under low pH conditions were performed. Following complex formation, the mixture was extensively diluted in PBS (28-fold dilution) to promote potential complex dissociation. Despite this dilution, the complex remained stable, even after recapture by protein-A followed by acidic elution (pH 3.6) and buffer exchange, with a calculated recovery yield of 70% (Figure S2).

### 4.3 Dissociation Rate Constants (*k*_*d*_) and Binding Affinity Analysis Using Surface Plasmon Resonance

We next assessed the binding and dissociation kinetics of TZM-Ecoil constructs to their corresponding complementary Kcoils using a surface plasmon resonance (SPR) assay, in which TZM-E5, TZM-E4, and TZM-E3 were injected onto complementary Kcoil-functionalized sensor chips (K5, K4, and K3, respectively). To minimize the potential for avidity effects, all sensor chips were functionalized with a low density of Kcoil peptides (15–40 resonance units (RU)) to ensure that two Ecoil tags within the same antibody were unlikely to simultaneously bind to two adjacent complementary Kcoil peptides on the chip surface.

Sensorgrams showing specific interactions between TZM-Ecoil constructs and their corresponding Kcoil peptides are presented in Figure 2A–C. The sensorgrams corresponding to binding of TZM-E5 to the K5 surface were characterized by a biphasic dissociation profile, consistent with previously published data (Figure 2A). A similar SPR profile was also observed for TZM-E4 (Figure 2B). In contrast, no detectable binding was observed for TZM-E3 to the K3-functionalized surface at the tested concentrations (Figure 2C), further supporting our UPLC-SEC results, once again underlying the poor affinity of the E3/K3 interaction. To quantify TZM-Ecoil/Kcoil complex stability, only data corresponding to the second phase of complex dissociation (*t* > 500s) were considered.^31^ The calculated apparent dissociation rate constants were 3.2 × 10^–5^ and 6.2 × 10^–5^ s^–1^ for TZM-E5- and TZM-E4, respectively. Based on our results from both UPLC-SEC and SPR analyses, we decided to exclude the E3/K3 complex from further analysis due to its insufficient affinity and stability.

**Figure 2:**
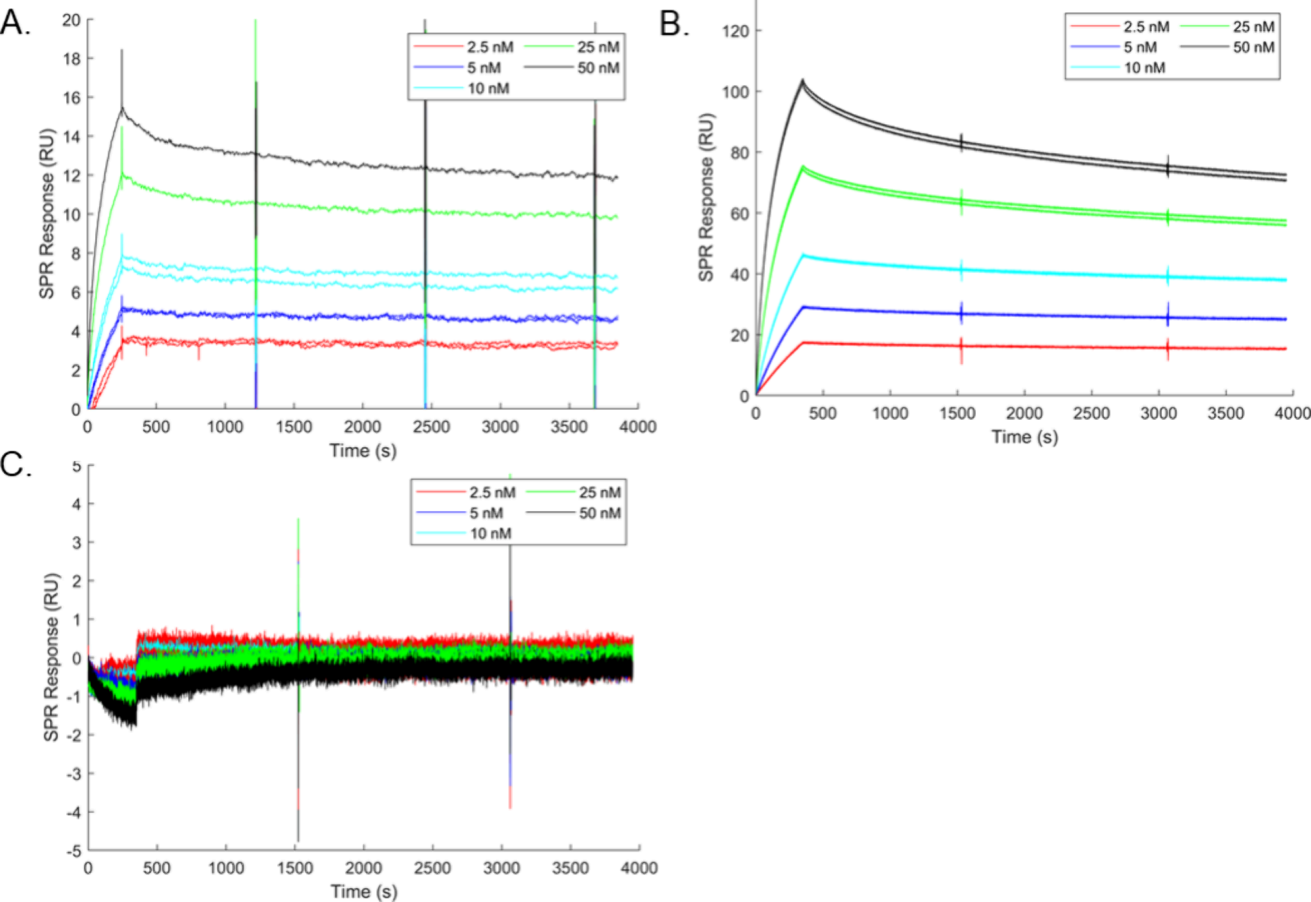
Interactions between the TZM-Ecoil constructs and the Kcoil surfaces. SPR sensorgrams corresponding to the interactions of TZM-E5 (A), TZM-E4 (B), and TZM-E3 (C), injected at concentrations ranging from 2.5 to 50 nM, onto K5-, K4-, and K3-functionalized sensor surfaces, respectively.

### 4.4 *In Vitro* Serum Stability

The stability of the E/K coiled-coil complex in the presence of serum was assessed by ELISA. TZM’s biological target, the human epidermal growth factor receptor 2 (HER2) extracellular domain, was coated on all plates. An HRP-conjugated rabbit anti-mRFP antibody was used to detect the TZM-Ecoil/Kcoil-mRFP complexes bound to HER2. The experimental conditions were optimized to minimize false positive signal due to the nonspecific binding of free (dissociated) Kcoil-mRFP components in the test (Figure S3). Complex stability (mediated by coiled-coil interactions) was also assessed in the presence of PBS alone. Lastly, the TZM-mRFP fusion construct was used as a control in the assay.

The TZM-mRFP fusion construct (Figure 3A, Table 1) demonstrated robust binding stability to HER2, with no significant loss of anti-mRFP-HRP signal observed over a 72 h incubation period in either serum or PBS. The calculated half maximal binding (EC_50_) value remained relatively constant (average 4.24 ± 1.37 ng/well) over a 72 h incubation period, independent of the buffer (PBS vs FBS) in which incubation was performed. Similarly, the noncovalent TZM-E5/K5-mRFP complex (Figure 3B, Table 1), maintained a stable apparent EC_50_ value (average 5.84 ± 0.61 ng/well), comparable to that of TZM-mRFP fusion. This indicates that the binding of TZM-E5 to the HER2 antigen, as well as the coiled-coil interaction between TZM-E5 and K5-mRFP, remains stable in serum and PBS for up to 72 h. In contrast, the E4/K4-mediated complex showed significant dissociation over time (Figure 3D), with apparent EC_50_ values varying from 30.2 ng/well at *T*_0_ to 650 and 1463 ng/well after 72 h in PBS and FBS, respectively. This instability suggests that the E4/K4 interaction was weaker than the E5/K5 interaction, i.e., that K4-mRFP significantly dissociated from TZM-E4 during the washing steps (*T*_0_) as well as during incubation in PBS and FBS. As the primary objective of the serum stability test was to prioritize the most suitable candidates for *in vivo* studies, we further concentrated our efforts on the E5/K5 complex only.

**Figure 3:**
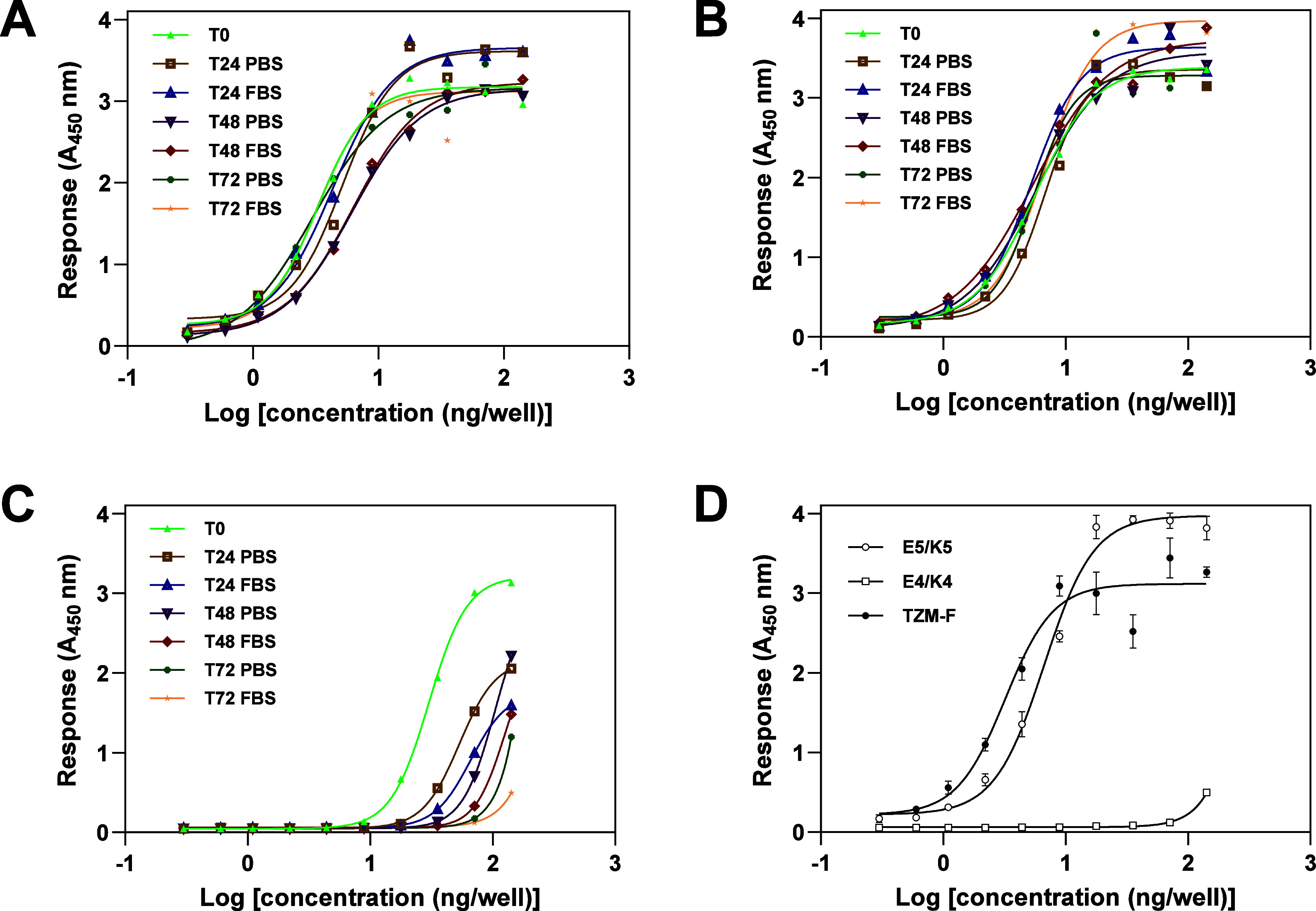
Stability of E/K coiled-coil complexes in serum or PBS. Dose–response curves of various TZM constructs binding to immobilized HER2 in the presence of serum (FBS) or PBS after incubations of 0, 24, 48, or 72 h. TZM-mRFP fusion (A); TZM-E5/K5-mRFP complex (B); TZM-E4/K4-mRFP complex (C); dose–response curves in FBS at 72 h for all types of complexes are shown in (D). In all cases, dose–response curves were fitted to a nonlinear log (agonist) vs absorbance using a four-parameter logistic function (*R* ^2^ ≥ 0.95). Data represent the mean of triplicate independent experiments.

**Table 1:** Calculated Half Maximal Binding (EC_50_) Values (ng/well) of TZM Constructs to HER2 at Various Timepoints in FBS or PBS

sample	*T*_0_	*T*_24_ PBS	*T*_24_ FBS	*T*_48_ PBS	*T*_48_ FBS	*T*_72_ PBS	*T*_72_ FBS
TZM-mRFP	3.32	5.18	4.36	6.13	6.29	2.97	3.20
TZM-E5/K5-mRFP	5.58	6.73	5.23	5.74	5.41	5.49	6.67
TZM-E4/K4-mRFP	30.2	53.4	66.1	105	126	650	1463

### 4.5 Animal Studies and Imaging

An Infrared (IR) fluorescence imaging study was conducted to assess the biodistribution, tumor targeting, and retention of the TZM-E5/K5-CF750 complex in both immunocompetent SKH1 and SKOV3 xenograft mouse models. TZM harboring four C-terminal E5 coils (HE5-LE5; TZM-E5) was complexed with the K5 complementary peptide, which was chemically conjugated to CF750 dye (K5-CF750) as a surrogate payload. For comparison, the parental TZM antibody was covalently conjugated to the CF750 dye (TZM-CF750) by using conventional lysine chemistry and used as a benchmark. Covalent labeling of TZM with CF750 was controlled in order to obtain ∼3.5–4 mol of CF750 dye per mole of TZM to mimic the labeling ratio of the TZM-E5/K5-CF750 complex. Additionally, a mixture consisting of conjugated K5-CF750 peptide and an equimolar amount of E5 peptide (denoted E5/K5-CF750) served as a negative control to evaluate nonspecific interactions.

First, the biodistribution of TZM-CF750, TZM-E5/K5-CF750 and E5/K5-CF750 was evaluated in immunocompetent SKH1 mice by dorsal and ventral imaging. The E5/K5-CF750 control showed rapid systemic distribution throughout the body within 5 min postinjection, with strong signals detected in the spinal cord, kidney, and the bladder (Figure S4). Increasingly high signal intensity in the kidneys and bladder indicated rapid renal clearance, as expected for small peptides.^42^ Rapid clearance of the signal from the body also indicated a low level of nonspecific binding. In contrast, the biodistribution of TZM-CF750 and TZM-E5/K5-CF750 differed significantly from the E5/K5-CF750 control. The covalently conjugated TZM-CF750 antibody exhibited rapid biodistribution throughout the whole body, which peaked after 24 h postinjection and persisted up to 336 h (Figure S5). In comparison, the whole-body signal for TZM-E5/K5-CF750 displayed body persistence up to 240 h (Figure S6). Similar to E5/K5-CF750 control, high signals were observed in the spinal cord, kidneys, and bladder, suggesting some dissociation of K5-CF750 peptide from the TZM-E5 antibody. Residual fluorescence analysis in organs collected at 336 h postinjection (Figures S4–S6) revealed distinct patterns. For E5/K5-CF750 and TZM-E5/K5-CF750, the highest signals were observed in the kidneys. However, the signal for TZM-E5/K5-CF750 was almost 2-fold lower than that of E5/K5-CF750 control. For the covalently conjugated TZM antibody, the highest signal was detected in the lungs, followed by the liver and kidneys.

Next, we conducted a similar biodistribution experiment in a SKOV3 xenograft mouse model. Figure 4 displays the dorsal imaging over time for mice injected with E5/K5-CF750 coiled-coil peptide control. This control construct demonstrated a rapid onset of whole-body signal, peaking within 5 min postinjection and persisting for at least 72 h. Notably, strong signals were detected in the spinal cord, kidneys, bladder, and tumor tissue as early as 5 min postinjection. As shown in Figure 4C, *ex vivo* imaging of residual fluorescence in harvested organs at 336 h postinjection revealed signal primarily in the kidneys, consistent with findings from a previous biodistribution study (Figure S4).

**Figure 4:**
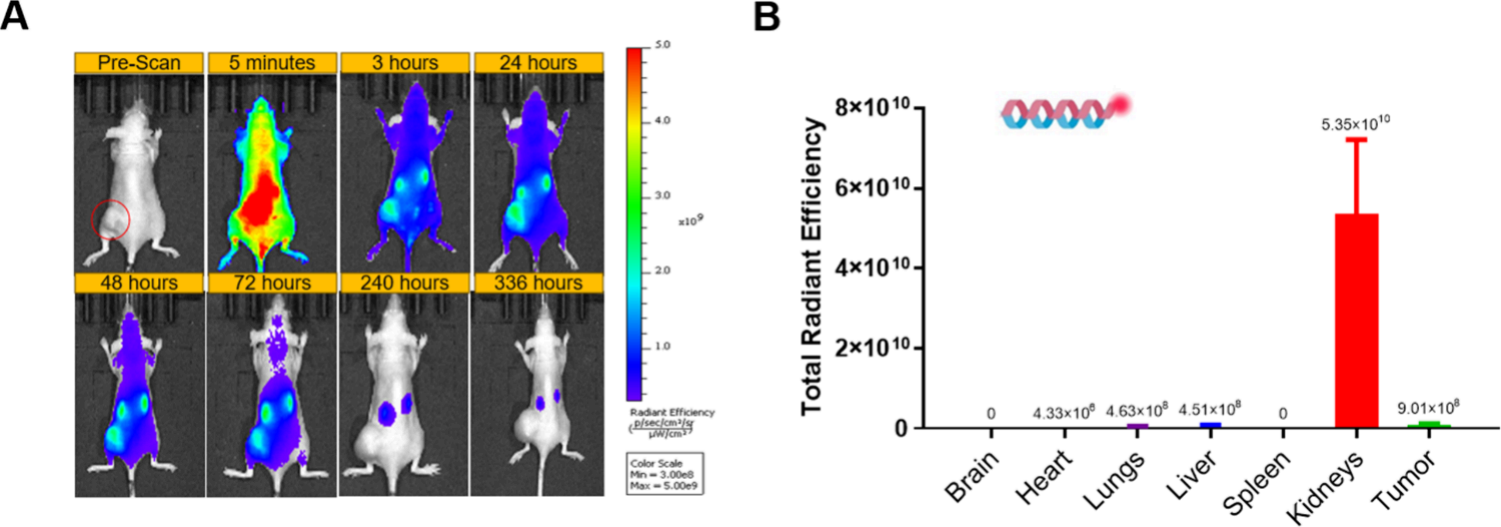
Biodistribution of E5/K5-CF750 intravenously injected in the SKOV3 xenograft mouse model evaluated. (A) *In vivo* dorsal fluorescent images of the whole mouse body captured at various time points to assess the biodistribution profile of E5/K5-CF750. (B) *Ex vivo* fluorescence measurements of total radiant efficiency conducted in various harvested organs at 336 h to evaluate residual retention of E5/K5-CF750.

For the covalently conjugated TZM-CF750, tumor tissue accumulation was detectable at 1 h postinjection, with transient signals observed in the liver and bladder between 5 min and 48 h (Figure 5A,B). Tumor accumulation remained high up to 336 h (14 days). *Ex vivo* fluorescence imaging of whole organs further confirmed this pattern, revealing a prominent signal localized within the tumor, with considerably weaker signals in the liver and kidneys, demonstrating the stability of covalently conjugated TZM-CF750 *in vivo* (Figure 5C).

**Figure 5:**
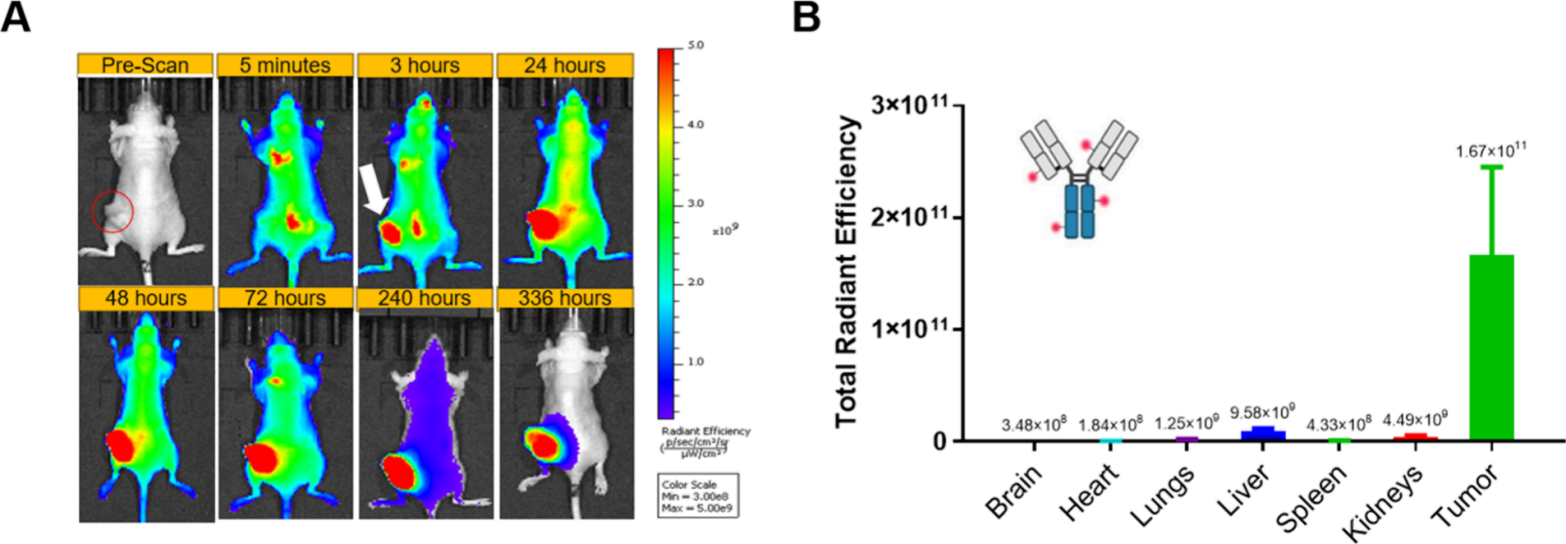
Biodistribution of TZM-CF750 intravenously injected in athymic nude/nude mice bearing SKOV3 xenografts. (A) *In vivo* dorsal fluorescent images of the whole mouse body captured at multiple time points postinjection to visualize the biodistribution profile of the covalently conjugated TZM-CF750. (B) *Ex vivo* fluorescence imaging measuring total radiant efficiency in various harvested organs at 336 h, to evaluate residual signal intensity across tissues.

The biodistribution profile of TZM-E5/K5-CF750 exhibited characteristics intermediate between those of E5/K5-CF750 and TZM-CF750. Indeed, as shown in Figure 6, whole-body signal was observed as early as 5 min postinjection, indicating rapid systemic distribution. Similar to the TZM-CF750 positive control group, a significant tumor-associated signal was detected within 3 h postinjection, which persisted for up to 240 h, demonstrating effective tumor retention. However, and similar to E5/K5-CF750, a high signal was also observed in the kidneys, indicative of renal clearance. This contrasted with TZM-CF750, where the liver and kidneys only exhibited low signal intensity. The distinct biodistribution profile of TZM-E5/K5-CF750 suggests partial dissociation of K5-CF750 peptide from the TZM-E5 antibody early after injection.

**Figure 6:**
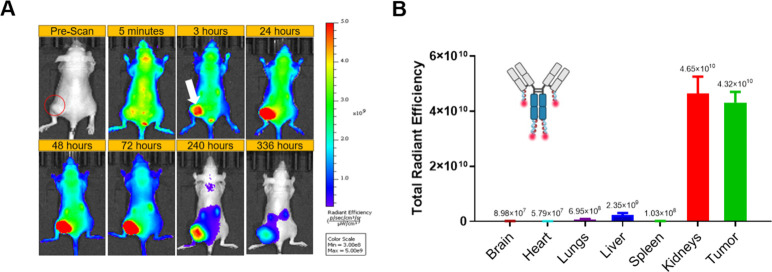
Biodistribution of TZM-E5/K5-CF750 intravenously injected in athymic nude/nude mice bearing SKOV3 xenografts. (A) *In vivo* dorsal fluorescent images of the whole mouse body at multiple time points to evaluate biodistribution profile of the construct. (B) *Ex vivo* fluorescence imaging to measure total radiant efficiency in various harvested organs at 336 h.

Fluorescence accumulation kinetics within the SKOV3 xenograft tumor model are shown in Figure 7A. The data indicate that the covalently conjugated TZM-CF750 exhibited the highest tumor accumulation, consistent with the strong signal intensity observed in the tumor region (Figure 5). Specifically, while TZM-CF750 displayed the highest intensity, TZM-E5/K5-CF750 showed an area under curve (AUC) of 4.4 × 10^13^, nearly half of the value observed for TZM-CF750, in accordance with the biodistribution data shown in Figure 6. In contrast, the E5/K5-CF750 control demonstrated the lowest AUC at 5.9 × 10^12^, one log lower than that of TZM-E5/K5-CF750, consistent with its rapid accumulation in the kidney, likely indicative of its blood clearance (Figure 4).

**Figure 7:**
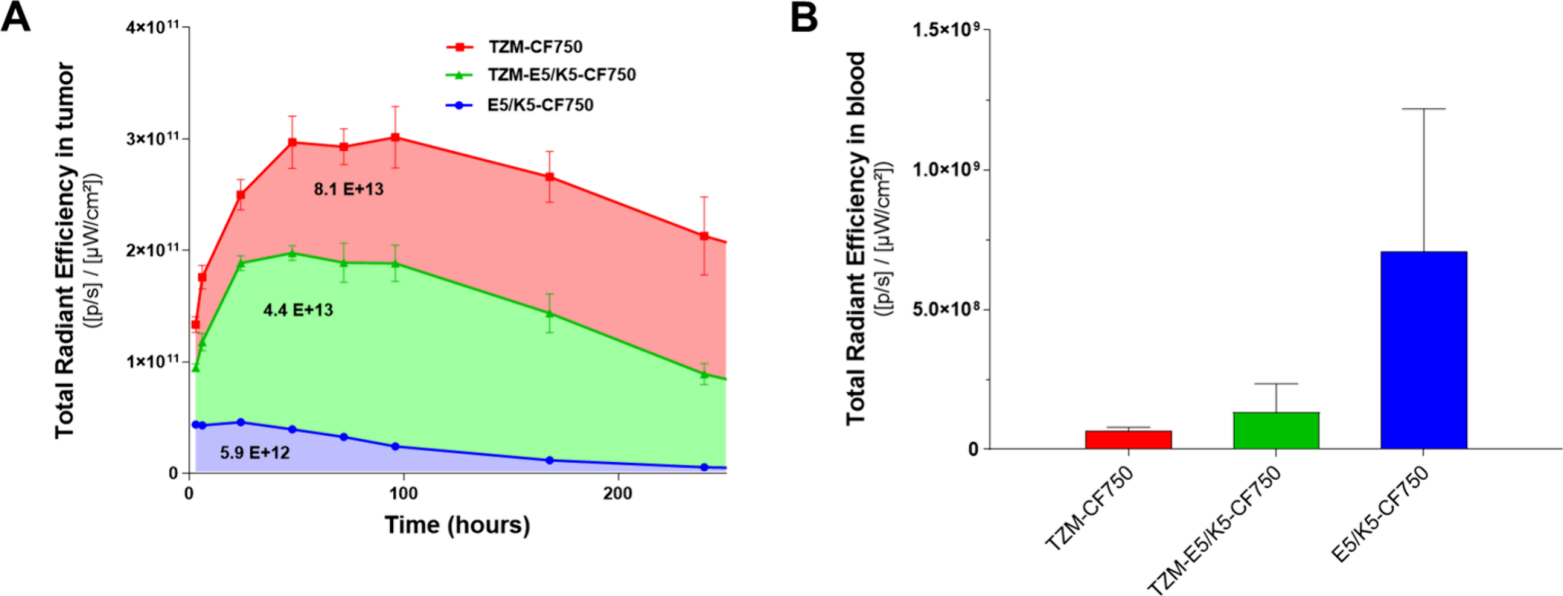
Total radiant efficiency in mice subcutaneously implanted SKOV3 tumors and in blood. (A) Fluorescence signals were collected in three separate animals at each time points after intravenous injection of the constructs. Values under each curve represent integrated area under the curve between 3 and 336 h (AUC3–336, [p/s] × h /[uW/cm^2^]) as determined using GraphPad Software (AUC: area under the curve; p: photon; s: second; μW: microwatts; cm^2^: area of the tumor ROI). (B) Total fluorescent signal measured in blood samples at 336 h. Each bar represents average values ± SD (*n* = 3).

Finally, the fluorescence intensity present in the blood at 336 h postinjection was assessed (Figure 7B). Mice injected with TZM-CF750 and TZM-E5/K5-CF750 displayed the lowest intensity of CF750 dye in their blood samples at 336 h (6.7 × 10^7^ and 1.3 × 10^8^, respectively) while the mice injected with E5/K5-CF750 test sample displayed 5–10 times higher blood fluorescence (7.1 × 10^8^).

## Discussion

Antibody-drug conjugates (ADCs) have recently regained significant interest in the oncology landscape, following notable success in their ability to treat cancer patients.^11^ The most important factors in increasing ADC safety in recent years have been improvements in conjugation technology, particularly site-specific conjugation, combined with enhanced control over the DAR and linker stability *in vivo.*

In this context, our study explored the potential of leveraging the high-affinity and high-specificity E-K-coiled-coil peptide interaction to develop a novel antibody conjugate manufacturing platform. We investigated the manufacturability and stability of a panel of Ecoil-tagged trastuzumab constructs combined with their complementary Kcoil peptide-conjugated surrogates. In addition, we assessed the *in vivo* biodistribution in immunocompetent mice and in a mouse xenograft tumor model. Results from these studies revealed the usefulness of the TZM-E5/K5-CF750 construct for tumor imaging and potential therapeutic use as an ADC. The E/K coiled-coil approach offers significant manufacturing advantages: (i) the antibody-drug complex could be conveniently assembled at the patient’s bedside by simply mixing the antibody-Ecoil construct with the Kcoil-drug moiety, and (ii) this method allows for the manufacturing of only one Kcoil-drug conjugate that can be paired with various antibody-Ecoil constructs, enhancing versatility and simplifying production. Since Ecoil tags are expressed at the C-termini of heavy and light chains of the antibody, they are highly exposed and serve as sticky handles to efficiently capture preconjugated Kcoil peptides with high affinity. To investigate the interactions between TZM-Ecoil and their Kcoil-mRFP counterparts, we performed two distinct assays. First, we employed UPLC-SEC to study complex formation following their mixing in a 1:4 molar ratio (Figure 3). Successful complex formation was confirmed by the appearance of higher molecular weight peaks corresponding to the TZM-Ecoil/Kcoil-mRFP complex and the simultaneous disappearance of the lower molecular weight peaks corresponding to unbound TZM-Ecoil and Kcoil-mRFP molecules. Among the three Ecoil/Kcoil candidates tested, the E3/K3 combination was the only one that failed to form complexes. This lack of stability has also been reported in other studies and led to its exclusion from our study.^28,31^ Second, we measured the TZM-Ecoil/Kcoil dissociation kinetics by using SPR biosensing. Here, we observed that the number of heptad repeats plays a critical role in the interaction stability. Specifically, we found that the TZM-E4 (HE4-LE4) interaction with the K4 peptide exhibited half the stability (twice the dissociation rate constant) of the TZM-E5 against the K5 peptide, while no interaction was detected with the E3/K3 pair. In summary, these findings emphasize the critical importance of the number of heptad repeats to obtain a stable E-K coil interaction. The difference in stability became more evident during an *in vitro* serum stability test. The TZM-E4/K4-mRFP showed a marked increase in apparent EC_50_ values in the HER2 ELISA when incubated in FBS, in stark contrast to TZM-E5/K5-mRFP (Figure 3C, Table 1). We previously showed that adding a Ecoil tag to the TZM HC and/or LC C-terminus did not interfere with its ability to bind to HER-2.^31^ Consistent with this, the comparable apparent EC_50_ values between the TZM-mRFP fusion protein and the TZM-E5/K5-mRFP complex (Figure 3F, Table 1) confirm that the association of K5-mRFP with the E5 coil fused to TZM’s HC and LC C-termini does not affect its HER2-binding affinity.

We then investigated the *in vivo* stability and biodistribution of the TZM-E5/K5-CF750 complex in immunocompetent mice. For these studies, we substituted CF750 for mRFP as our initial experiments indicated that mRFP intrinsic fluorescence was not sufficient for *in vivo* imaging (data not shown). The E5/K5-CF750 coiled-coil peptide exhibited high signal intensity in the kidneys, consistent with rapid renal clearance (Figure S4), a characteristic of small peptidic compounds.^42^ Furthermore, the absence or weak signals observed in the other organs indicate that the E5/K5-CF750 peptide complex is not significantly retained in tissues due to nonspecific interactions. In contrast, the covalently conjugated TZM-CF750 antibody did not show any fluorescence accumulation in the kidney (Figure S5). Surprisingly, the TZM-E5/K5-CF750 complex displayed a significant signal in the kidneys at the early time points, similar to the E5/K5-CF750 complex (Figure S6), suggesting its partial dissociation or degradation, resulting in renal clearance of the free K5-CF750 peptide *in vivo*. This early loss of K5-CF750 could be related to the previously observed partial dissociation of K5-mRFP from the LC-E5 moiety of the TZM-E5 construct, as indicated by SPR.^31^ While the mechanism responsible for this early dissociation is currently unknown, similar *in vivo* payload losses have been reported for several approved ADCs.^26^ Finally, the weak signal found in lung and liver for TZM-E5/K5-CF750 and TZM-CF750 is in accordance with other studies using conjugated trastuzumab, suggesting a low level of HER2 expression in those tissues.^43–45^

Lastly, we examined the complex stability, tumor targeting, and retention of the tested constructs in a HER2-expressing SKOV3 xenograft mouse model. The E5/K5-CF750 controls exhibited a pharmacokinetic pattern similar to that observed in the biodistribution study (Figure 4), consistent with the behavior of molecules smaller than 65 kDa that undergo rapid systemic distribution and renal clearance.^46–48^ Furthermore, despite the initial accumulation of fluorescent signal in the tumor, likely due to enhanced local blood flow, minimal signal was detected at the tumor site between 96- and 336 h postinjection, suggesting the absence of nonspecific binding and relatively rapid clearance from the tumor. The covalently linked TZM-CF750 construct did not show any apparent dissociation or kidney accumulation and efficiently targeted the tumor (Figure 5), demonstrating superior stability and retention. In contrast, the TZM-E5/K5-CF750 complex displayed a more complex profile. While some early dissociation was evident in the bloodstream and as seen in the immunocompetent mice, the coiled-coil interaction was sufficiently stable to last in the blood circulation and to reach the tumor site (Figure 6). This interaction remains stable enough to be detected at the tumor site for up to 10 days, but at a level somewhat lower compared to the TZM-CF750 construct.

The data presented in this study thus offer compelling evidence that while the TZM-E5/K5-CF750 complex exhibits moderate early dissociation, which should be addressed in future designs, its stability remains adequate to achieve meaningful tumor retention, offering a viable platform for ADC development with potential manufactory advantages. Contrary to conventional conjugation techniques, which require subsequent purification steps to eliminate residuals or undesired species,^19,49^ our E/K-based strategy enables a simplified ″single-step″ noncovalent conjugation procedure that is achieved by simply mixing an antibody-Ecoil with a payload-carrying Kcoil. This approach would significantly improves ADC manufacturing by (i) greatly simplifying the conjugation process, (ii) reducing the adverse effects that chemical conjugation reactions can have on antibody functionality and bioactivity,^50^ (iii) eliminating the need for manufacturing GMP-grade enzymes (e.g., sortases, transglutaminases or formylglycine-generating enzymes) and their subsequent removal when chemo-enzymatic approaches are used for ADC production^51^ and (iv) reducing heterogeneity of the final ADC product. The use of an accurate stoichiometric mixture of mAb-Ecoil and Kcoil-drug would allow for the generation of ADCs with tunable DARs depending on the number and positioning of Ecoil tags on the antibody’s light/heavy chains, enabling the production of more homogeneous ADC populations.

Additionally, the rapid association kinetics of E/K coiled-coil interactions make our strategy compatible with a ″bedside formulation″ approach. That is, the Kcoil payload and Ecoil-fused monoclonal antibody may be produced, formulated, and vialed separately, then simply mixed together for less than an hour prior to administration. Hence, our approach may eliminate some of the therapeutics’ shelf life limitations while enabling on-demand customization of ADCs to be tailored to individual patient needs. This is particularly beneficial for payloads like radionuclides with limited half-lives,^52,53^ as it simplifies their manufacturing and maximizes their therapeutic efficacy and cost-effectiveness.

While we demonstrated the efficient binding, accumulation, and persistence of E/K coiled-coil conjugated trastuzumab at the tumor site, the mechanism behind the partial but undesired early *in vivo* “release” of the surrogate payload needs to be understood and resolved. In addition, effective payload delivery into tumor cells may be another important aspect for a potent ADC. The nature of the linker often dictates how, where, and when the cytotoxic payload is released within the target cells. Both plasma stability and efficient release of the active drug at the tumor site or upon ADC internalization in the tumor cells are two critical aspects of linkers.^54,55^ Since it has been shown that the heterodimeric E/K coiled-coil interaction undergoes homodimerization under low acidic pH conditions,^27,56^ this could potentially favor release of the payload of an E/K coiled-coil-based ADC once it reaches the endosomal-lysosomal system. The release of the drug may also be triggered through enzymatic cleavage/degradation by proteases.^57,58^ Further investigation is necessary to evaluate whether TZM-E5/K5-CF750 is internalized and releases its surrogate drug in tumor cells.

## Conclusions

Our study demonstrated for the first time the potential of coiled-coil interactions for the development of antibody conjugates such as ADCs, where a K5 coil that is conjugated to a payload can be tethered to a monoclonal antibody-E5 coil fusion simply by mixing both components together without any additional chemistry or purification step involved. We showed robust complex formation following mixing the TZM-E5 with K5 conjugate and demonstrated its ability to reach and accumulate at the tumor site. However, the observed early dissociation of the fluorescent payload *in vivo* calls for further optimization of the Ecoil/Kcoil interaction to increase its *in vivo* stability. This is a critical aspect to ensure the safety and efficacy of an eventual coiled-coil-based ADC. Also, further investigation is necessary to determine if the drug moiety is released after antibody conjugate internalization at the target site, since this release is often required for the drug to exacerbate its cellular toxicity.^59^ Overall, this study paves the way toward the development of a novel and robust antibody conjugate manufacturing platform that offers great potential for therapeutic and diagnostic uses.

## Supplementary Material



## Data Availability

Data presented in this manuscript is available upon reasonable request to the corresponding author.

## Notes

(Figure S1) mRFP and Kcoil-mRFP production; (Figure S2) UPLC-SEC chromatograms of TZM-E5/K5-mRFP complex prior to or after ∼30-fold dilution in PBS and recapture by protein-A; (Figure S3) validation of the serum stability ELISA; (Figure S4) biodistribution study of E5/K5-CF750 test sample; (Figure S5) biodistribution study of the TZM-CF750 sample; (Figure S6) biodistribution study of the TZM-E5/K5-CF750 sample; (Table S1) UPLC-SEC-MALS analysis of TZM, TZM-mRFP, TZM-Ecoils, Kcoils-mRFP, and E/K coiled-coil complexes (PDF)
