# Supplementary material for: Use of Coiled-Coil Affinity Peptides to Manufacture Antibody Conjugates
Source: Bioconjug Chem. 2025 Jul 29;36(8):1670–82. doi: 10.1021/acs.bioconjchem.5c00178 (PMC12371696; doi:10.1021/acs.bioconjchem.5c00178)
Supplement: Supplementary file 1 [file bc5c00178_si_001.pdf]

SUPPLEMENTARY INFORMATION

Use of Coiled-Coil Affinity Peptides to Manufacture Antibody Conjugates

Seyed Farzad Baniahmad [a, b], Alina Burlacu [b], Laurence Delafosse [b], Mauro Acchione [b], Miriam Simmons [b], Binbing Ling [c], Umar Iqbal [c], Maria J Moreno [c], Gregory De Crescenzo [d], Yves Durocher [a, b, e, †]

[a] *Department of Biochemistry and Molecular Medicine, Faculty of Medicine, University of Montreal, Montreal, Québec, Canada.*

[b] *Human Health Therapeutics Research Centre, Building Montreal-Royalmount, National Research Council Canada, Montréal, Québec, Canada.*

[c] *Human Health Therapeutics Research Center, National Research Council Canada, Ottawa, Ontario, Canada.*

[d] *Department of Chemical Engineering, Polytechnique Montréal, Montréal, Québec, Canada*

[e] *PROTEO: The Quebec Network for Research on Protein Function, Structure, and Engineering, Université du Québec a Montréal, Québec, Canada*

† Corresponding author: yves.durocher@nrc.gc.ca

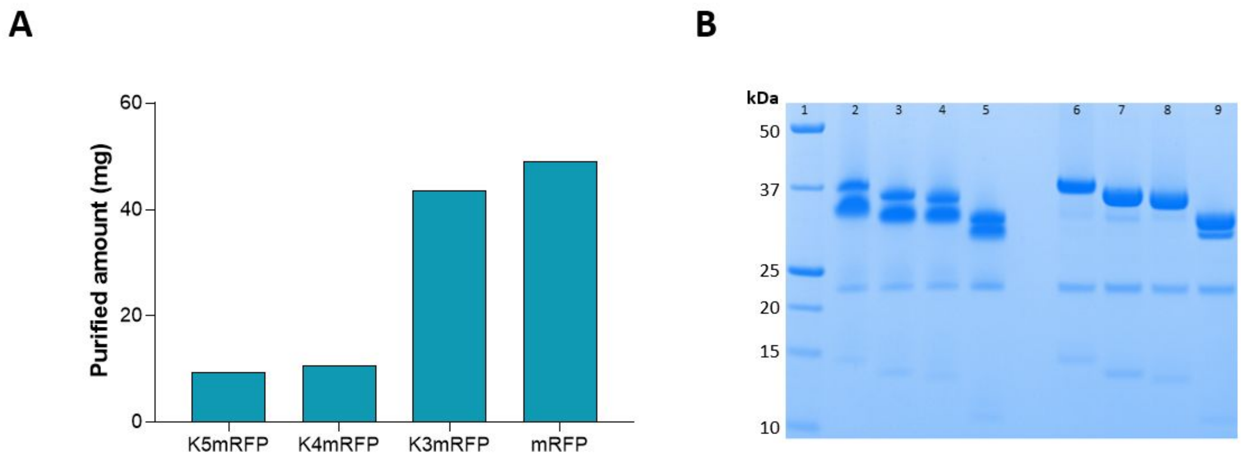

Figure S1. mRFP and Kcoil-mRFP production. (A) IMAC purification yields for mRFP and Kcoil-mRFP constructs (from 250mL transient gene expression in CHO-3E7 cells). (B) SDS-PAGE characterization of purified mRFP and Kcoil-mRFP constructs under non-reducing (lanes 2-5) and reducing conditions (lanes 6-9). Lane 1: molecular weight ladder. Lane 2: non-reduced K5-mRFP. Lane 3: non-reduced K4-mRFP. Lane 4: non-reduced K3-mRFP. Lane 5: non-reduced mRFP. Lane 6: reduced K5-mRFP. Lane 7: reduced K4-mRFP. Lane 8: reduced K3-mRFP. Lane 9: reduced mRFP.

Investigating the stability of TZM-E5/K5-mRFP following dilution in PBS and recapture on protein-A

We investigated the stability *TZM-E5/K5-mRFPs complex* following a ~30-fold dilution in PBS. To do so, 4 nmol of HE5/LE5 (TZM-E5) antibody was mixed with 16 nmol of K5-mRFP (in a total volume 364 µl). The mix was incubated for 30 minutes at room temperature, then 50 µl was kept aside for UPLC-SEC analysis (Sample A; Fig. S2, panel A). The rest of the mix was diluted in 10 ml of Dulbecco's Phosphate Buffered Saline (PBS; Hyclone) and incubated for 120 minutes at room temperature. Then, 50 µl of the sample was kept aside for UPLC-SEC analysis (Sample B; Fig. S2, panel B). After the 120 minutes incubation, the diluted mix was incubated with 100 µl of MabSelect SuRe (GE Healthcare, cat# 17–5438–02) resin equilibrated in PBS. The resin was washed with PBS, and the antibody was eluted with 500 µl of 100 mM citrate buffer pH 3.6. The eluted antibody was buffer-exchanged with 500 µl of PBS using CentriPure P-5 desalting columns (emp Biotech GmbH, Germany), and 50 µl of the sample was kept aside for UPLC-SEC analysis (Sample C; Fig S2, panel C).

UPLC-SEC analysis conducted using a 4.6 × 150 mm BEH200 SEC column with 1.7 µm particle size (Waters, Milford, MA) connected to an Acquity H-Class Bio UPLC system (Waters) with a photodiode array (PDA) detector. Chromatography was performed in a mobile phase (0.2 M potassium phosphate,

0.2 M potassium chloride, 0.02% Tween 20, pH 7.0) at 30°C at a flow rate of 0.4 mL/min. Measurement of the integrated areas and determination of the retention time (RT) for peaks at 280 nm were performed using Empower™ 3 software (Waters, Milford, MA).

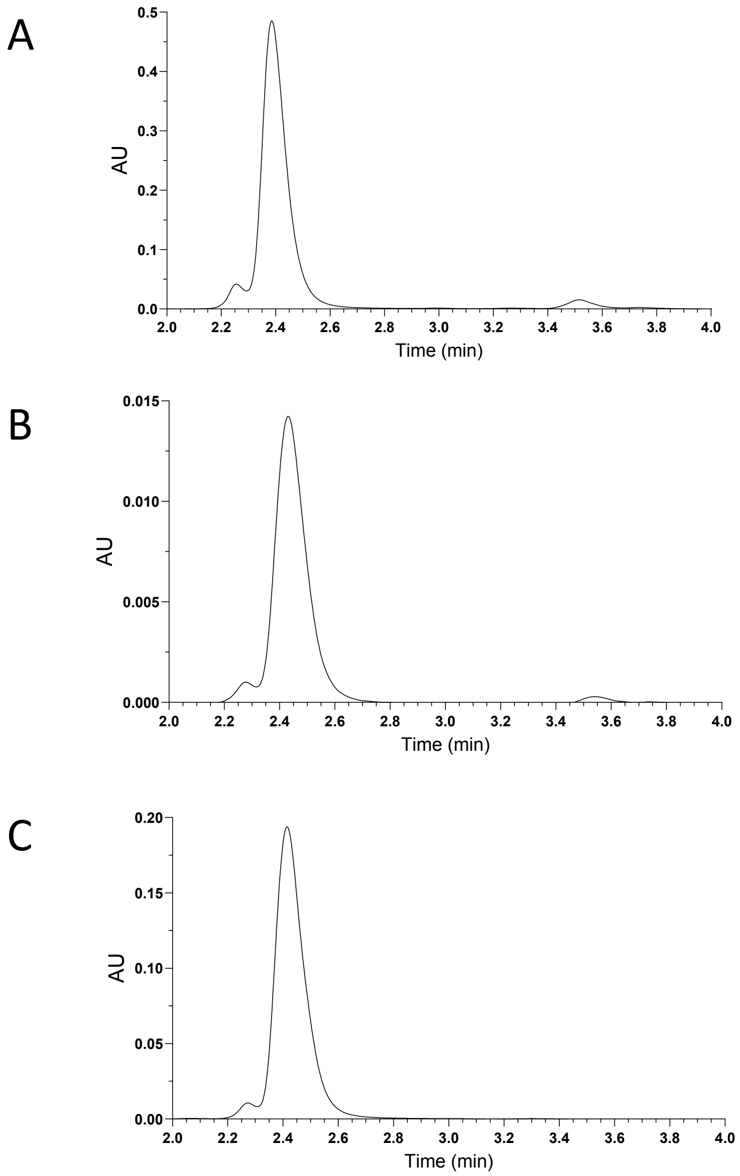

Figure S2. UPLC-SEC chromatograms of TZM-E5/K5-mRFP complex prior to or after ~30-fold dilution in PBS and recapture by protein-A. (A) Undiluted TZM-E5 + K5-mRFP mixture after 30 min incubation (Sample A); (B) Mixture diluted ~30-fold in PBS and incubated for 120 min (Sample B); (C) Recovery of diluted mixture (Sample B) by Protein-A followed by acid elution and buffer exchange in PBS (Sample C).

Investigating specificity and binding stability of TZM-E5/K5-mRFP in the HER2 ELISA assay

This experiment was performed to assess non-specific binding of K5-mRFP in an ELISA assay, compared to the TZM-E5/K5-mRFP complex. When using the TZM-E5/K5-mRFP complex, binding to HER2 was detected as shown by the robust dose-response curve obtained after detection with anti-mRFP-HRP antibody (Fig. S3, panels A and B). However, when using TZM-E5 (panel A) or K5-mRFP (panel B) alone, no signal was observed, indicating absence of non-specific binding of anti-mRFP to TZM-E5 or non-specific binding of K5-mRFP to the surface. The HER2 binding stability and non-specific binding verification of TZM-fusion, TZM-E5/K5-mRFP complex, and K5-mRFP under varying wash conditions (2x, 3x, 4x, 5x washes). The results show that no signal is observed for K5-mRFP, indicating no binding and confirming the absence of false positives. The binding stability of TZM-mRFP (B) and TZM-E5/K5-mRFP (C) test samples to HER2 over PBS wash cycles was assessed. No reduction in the dose-response signal was observed over 5 wash cycles, indicating that the binding of TZM to HER2 is stable and that the coiled-coil complex does not dissociate.

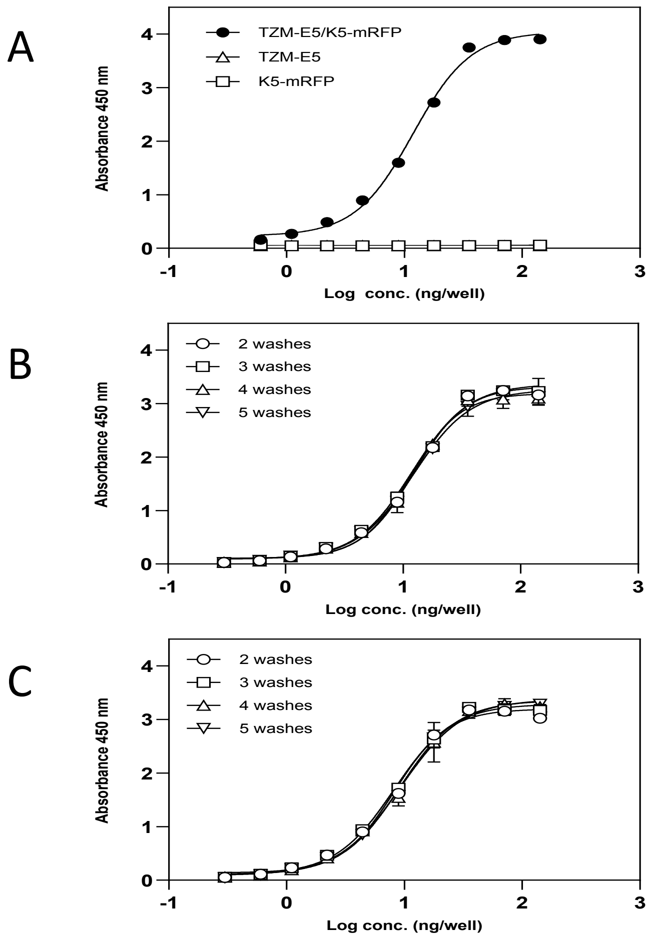

Figure S3. Validation of the serum stability ELISA (A) Binding interactions of TZM-E5/K5-mRFP (full circles), TZM-E5 (empty triangles) and K5-mRFP (empty squares) test samples on HER2 surfaces to detect non-specific binding. ELISA plates were incubated with the test samples, washed with PBS and then incubated with anti-mRFP-HRP antibody. No signal is observed for TZM-E5 or K5-mRFP indicating absence of non-specific binding of anti-mRFP-HRP antibody to TZM-E5, or K5-mRFP to HER2 surface, respectively. Binding stability of TZM-E5/K5-mRFP (panel B) and TZM-mRFP (panel C) to HER2 surface over wash cycles with PBS (2x, 3x, 4x, 5x washes).

E5/K5-CF750 biodistribution study in an immunocompetent SKH1 mouse model

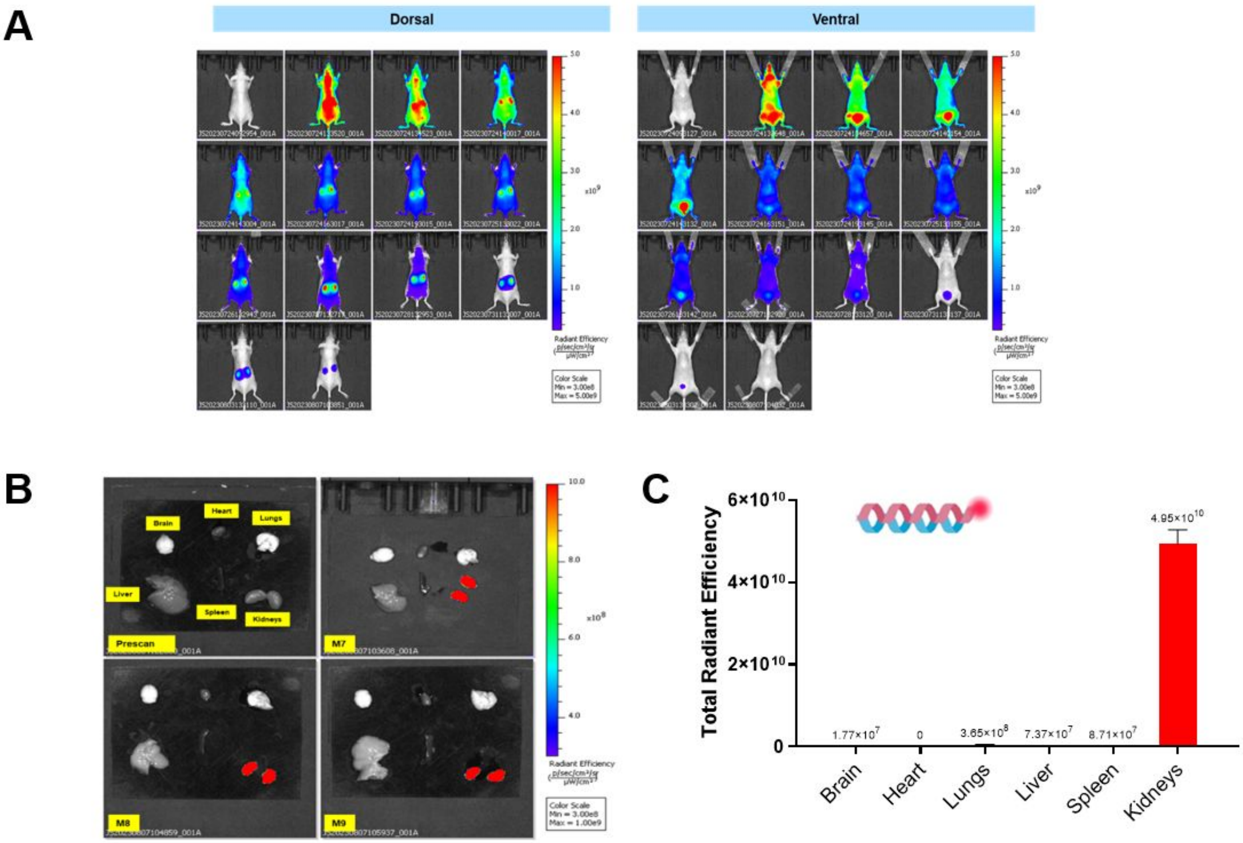

Figure S4. Biodistribution study of E5/K5-CF750 test sample. (A) In vivo fluorescence kinetic imaging of immunocompetent SKH1 mouse injected with. E5/K5-CF750 (B) Fluorescence imaging of various organs at 336 hours. (C) Total radiant efficiency in various organs at 336 hours.

TZM-CF750 biodistribution study in an immunocompetent SKH1 mouse model

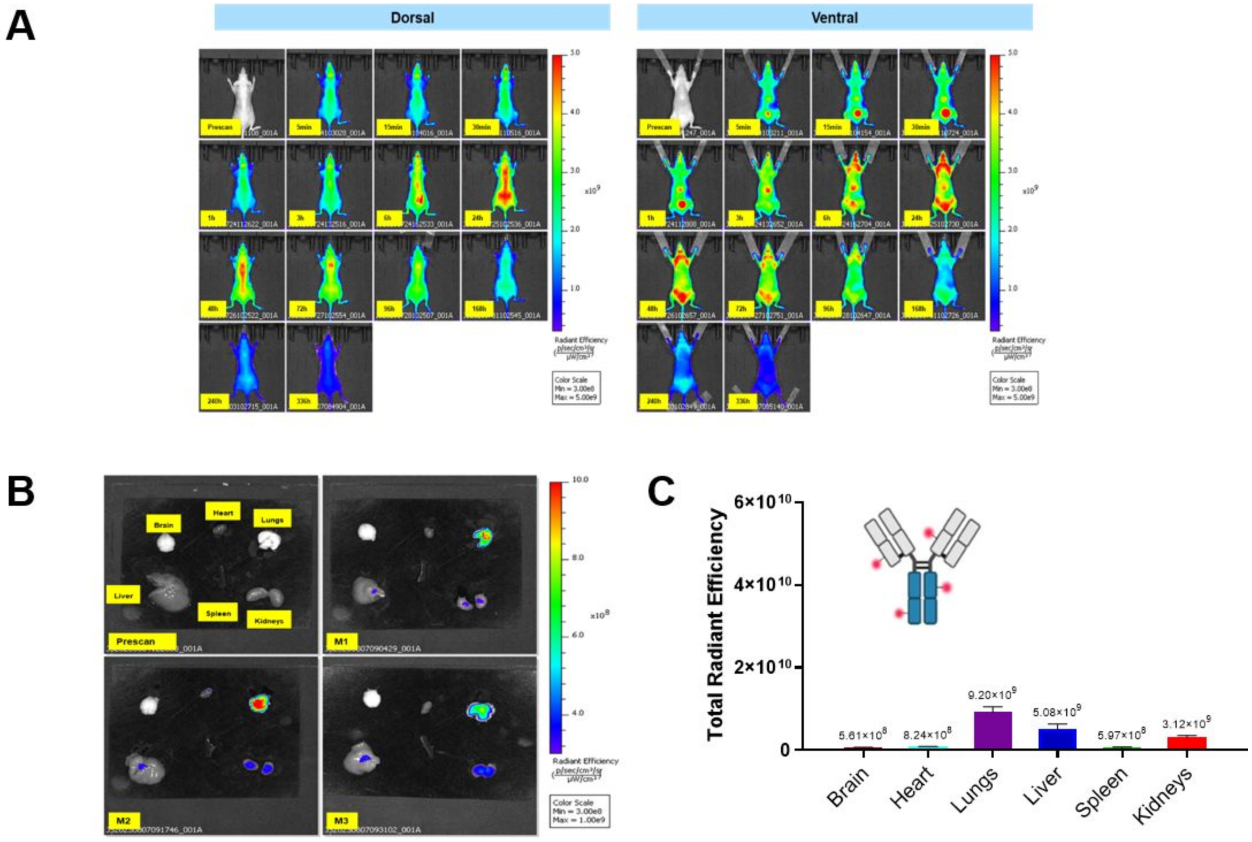

Figure S5. Biodistribution study of TZM-CF750 sample. (A) In vivo fluorescence kinetic imaging of immunocompetent SKH1 mouse injected with covalently conjugated TZM-CF750. (B) Fluorescence imaging of various organs at 336 hours. (C) Total radiant efficiency in various organs at 336 hours.

TZM-E5/K5-CF750 biodistribution study in an immunocompetent SKH1 mouse model

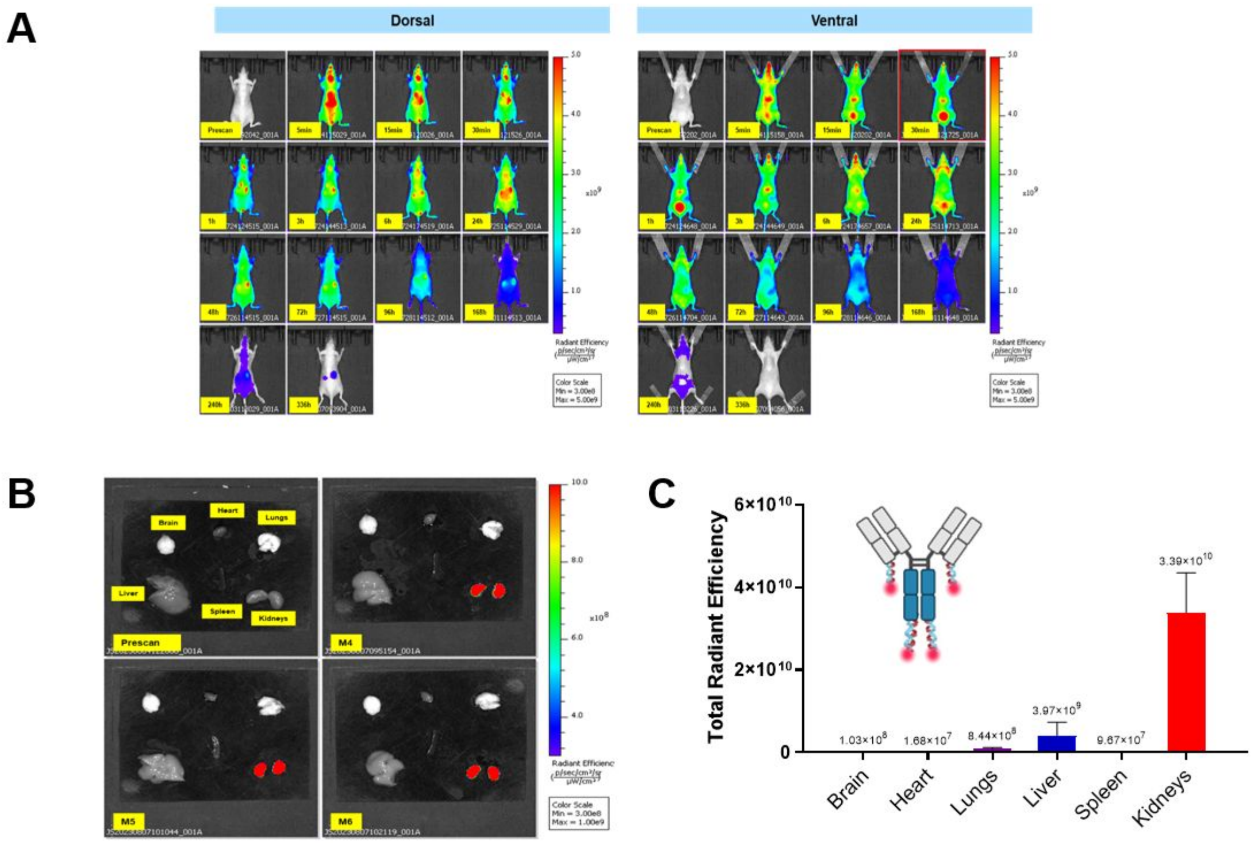

Figure S6. Biodistribution study of TZM-E5/K5-CF750 sample. (A) In vivo fluorescence kinetic imaging of immunocompetent SKH1 mouse injected with TZM-E5/K5-CF750 test sample. (B) Fluorescence imaging of various organs at 336 hours. (C) Total radiant efficiency in various organs at 336 hours.

Table S1: UPLC-SEC-MALS analysis of TZM, TZM-mRFP, TZM-Ecoils, Kcoils-mRFP and E/K coiled-coil complexes (Complementary MALS data for Figure 3 chromatograms).

**Construct**	**Peak** (min)	**M_{MALS}** (kDa)	**Theoretical M_w** (kDa)
**TZM**	3.06	139	145
**TZM-mRFP**	2.54	314	263
**TZM-E5 (HE5-LE5)**	2.66	158	165
**K5-mRFP**	3.37	169	32.7
**TZM-E5 / K5-mRFP**	2.41	338	296
**TZM-E4 (HE4-LE4)**	2.75	155	162
**K4-mRFP**	3.65	167	31.5
**TZM-E4 / K4-mRFP**	2.64	244	288
**TZM-E3 (HE3-LE3)**	2.80	155	159
**K3-mRFP**	3.69	174	30.9
**TZM-E3 / K3-mRFP**	2.80	155	283
